# A prospective observational study assessing the feasibility of measuring blood lead levels in New Zealand hunters eating meat harvested with lead projectiles

**DOI:** 10.1016/j.conctc.2017.02.002

**Published:** 2017-02-08

**Authors:** Eric J. Buenz, Gareth J. Parry, Brent A. Bauer, Lauren M. Matheny, Klaasz Breukel

**Affiliations:** a Nelson Marlborough Institute of Technology, Nelson, New Zealand; b University of Minnesota, Minneapolis, MN, United States; c Mayo Clinic, Rochester, MN, United States; d LR Clinical and Statistical Consulting, Denver, CO, United States

**Keywords:** Heavy metals, Toxicology, Deer

## Abstract

There is no safe level of lead exposure. Correlations suggest that hunters harvesting wild game with lead bullets may be at risk of lead exposure through eating minute lead particles from shrapnel in their wild game. This feasibility study will determine if it is possible to conduct an interventional controlled, blinded study to evaluate if there is a causal relationship between meat harvested with lead bullets and elevated blood lead levels in those who consume the meat. This is an observational case crossover study and the primary outcome is blood lead levels. Individuals will have blood lead levels measured 2–4 days after eating one serving of meat harvested with lead bullets. At three potential washout periods these same individuals will have a subsequent blood lead level analysis. This observational study will provide the data necessary to determine the washout period and sample size for a prospective interventional study to evaluate if meat harvested with lead bullets raises blood-lead levels in those who consume the meat. This study has been approved by the Health and Disabilities Ethics Committees of New Zealand.

**Trial registration:**

NCT02775890.

## 1 Introduction

As lead is one of the few substances that does not have a safe level of exposure, it is important to minimize all exposure [1], [2]. Hunters can use lead or lead-free bullets [3], and a vast majority choose to use lead bullets [4], [5], primarily because of tradition and the belief that lead bullets are safe. Non-lead projectiles are considered premium ammunition [6], but they are not significantly more expensive in some world areas [7]. There is a correlation between hunters using lead projectiles and elevated lead blood levels [8], [9], [10], however a causal link is unestablished [11]. As lead-shot meat is sold globally, this potential lead contamination is a global health concern [12].

This is a clinical research protocol to determine the feasibility of assessing lead levels in hunters who use lead projectiles. This observational study will determine if it is possible to perform a subsequent interventional blinded controlled study of lead levels in hunters using lead or lead-free projectiles following consumption of self-harvested wild game. The hypothesis for the subsequent study is that minute lead particles from shrapnel dispersed through the animal during harvest are ingested and this exposure results in increased lead blood levels [3]. This current observational study will establish the sample size and washout period for the subsequent interventional study. This observational study will be conducted in compliance with the protocol, Good Clinical Practice Standards, associated regulations and institutional research requirements.

There are three biological compartments where unexcreted lead is stored: blood, soft tissue, and mineralising tissues. After absorption, lead is stored in the blood compartment with a half-life of 28 [13] – 36 [14] days. Lead rapidly disperses into soft tissues post-exposure and in soft-tissues it has a half-life of approximately 40 days [15]. The primary long term repository of lead in the body is mineralizing tissue in bones and teeth [16]. Importantly, these mineralizing tissue stores can be mobilized and increase lead levels in the blood. The long half-life of lead and the dynamic nature of storage with the various compartments present challenges in identifying causal relationships between lead exposure and blood lead levels.

**Fig. 1:**
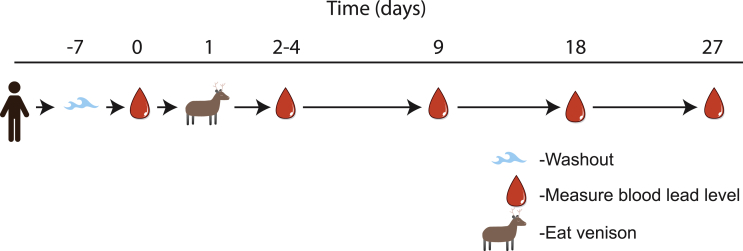
**Design of the current feasibility study**. Participants in the current study will harvest deer with standard lead projectiles. A baseline lead measure, a measure immediately after eating the lead-shot meat, and measures at 9, 18 and 27 days. These blood lead levels will allow a model to be built determining appropriate sample size for each potential washout period.

This current study establishes the parameters necessary to assess whether hunters eating meat shot with lead projectiles have elevated blood lead levels. For this feasibility study, hunters will be asked to abstain from consuming meat harvested with lead projectiles for 7 days. After 7 days, hunters will provide a baseline blood sample. Hunters will then consume meat that was harvested with lead projectiles and provide an additional blood sample 2–4 days (the blood-lead level peak post exposure [17]) after consumption. Hunters will also provide subsequent blood samples at periods of 9, 18, and 27 days. During the 27 day period, hunters will not consume meat harvested with lead projectiles. (Fig. 1). We have chosen to use deer as the species for this study to reduce variation; New Zealand is the ideal place to conduct this study because of year-round hunting of deer [18]. This study design will allow for the development of a conditional linear effects model, which will account for the inherent correlation of longitudinal data that involves repeated measures on the same subject over a period of time. A conditional model will also be more robust to any missing data that may arise through the course of the study, which may also facilitate a reduction in the required number of subjects necessary for this feasibility study.

**Fig. 2:**
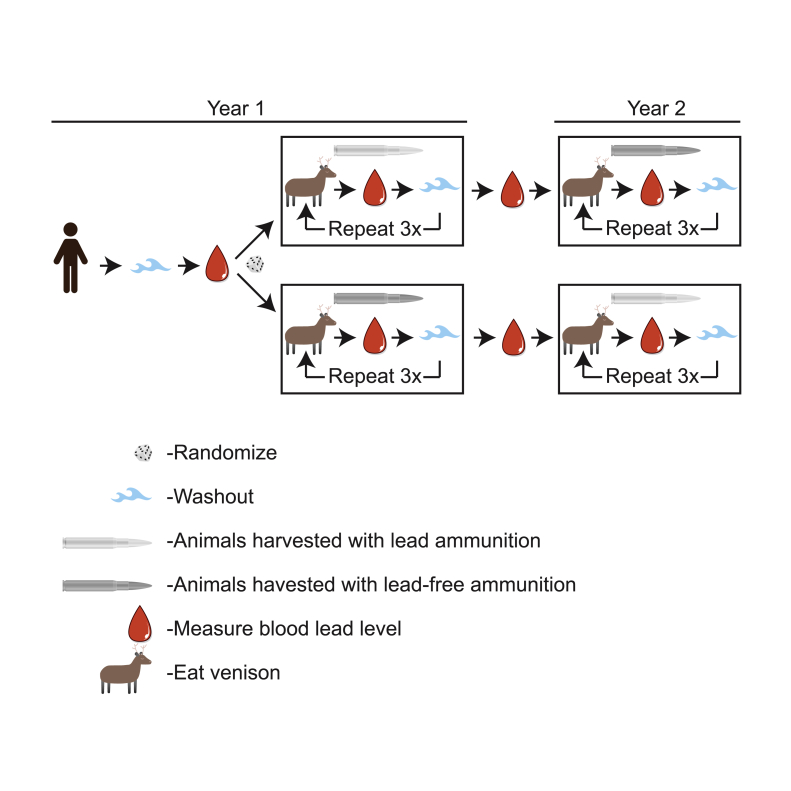
**Design of the proposed subsequent crossover study**. Participants in the subsequent study will be randomized to the lead or lead-free projectile arm of the study. After one year these participants will cross over to the other arm. Washout period and sample size will be determined by the results of the current feasibility study.

If this current observational study determines that an interventional study is feasible, the subsequent interventional study would randomize hunters into two groups: hunters using lead projectiles and hunters using non-lead projectiles. All hunters would undergo an initial wash-out period and then have baseline blood lead level recorded. Hunters in both cohorts would then eat the harvested meat and blood lead levels would be recorded 2–4 days after eating the self-harvested meat. Hunters would then repeat this process for a replication of 3 times during the year. After one year, the hunter would cross over to the other type of projectile and repeat the entire process again (Fig. 2). This case crossover study would be analyzed utilizing a conditional linear effects model to account for the correlative nature of longitudinal data involving repeated measures. Additionally, this model will allow for other factors to be included in the model, including time-dependent covariates. Additionally it would be possible to use isotope analysis to identify if the lead levels in the blood of participants were primarily from the environment or from the lead projectiles [10], [19]. Fortunately, baseline blood lead levels in New Zealand are similar to levels in other developed countries [20] thus allowing a broad extrapolation of the data generated.

## 2 Material and methods

### 2.1 Study objectives

The primary objective of this observational study is to determine the washout period and sample size for a subsequent study measuring lead levels in hunters in New Zealand after eating wild game shot with lead projectiles. This primary objective will be accomplished through measuring lead levels in hunters who have recently eaten meat harvested from deer killed with lead projectiles and subsequently measuring blood lead levels of those same hunters after abstaining from eating meat harvested from deer killed with lead projectiles (Fig. 1).

The secondary objective is to determine the feasibility of a blinded controlled interventional trial comparing blood lead levels in hunters using lead and lead-free projectiles for harvesting wild game for consumption. Specifically, this aspect of feasibility will be determined by participant compliance during this feasibility study.

### 2.2 General study design

There are two periods of analysis for this study.

1. Hunters that within 2–4 days prior to blood being drawn have eaten self-harvested wild deer shot with lead projectiles; and
2. Hunters that have abstained from eating self-harvested wild deer shot with lead projectiles for specific periods of time.

**Fig. 3:**
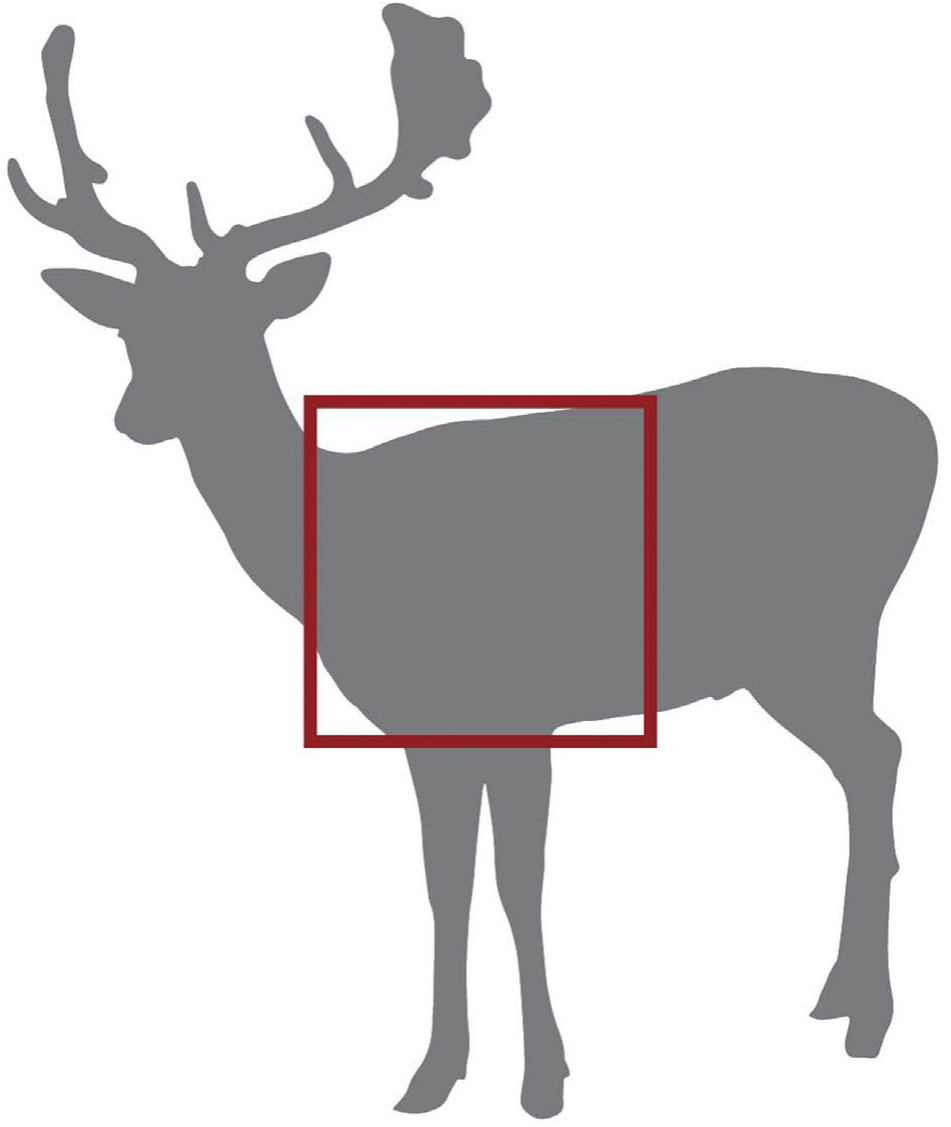
**Required shot placement for participation in the study**. Animals harvested for this study will be shot in the area indicated by a red box. This shot placement will be validated by photographs submitted to the study coordinator and will be the primary qualifying criteria for participating in the study. (For interpretation of the references to colour in this figure legend, the reader is referred to the web version of this article.)

Hunters in period 1 and period 2 are the same individuals; they will be consented for the study once they have harvested an animal and provide pictures of shot placement. In order to avoid bias, the investigator performing the data analyses and the laboratory staff will be blinded to the samples. The participants will know when they are submitting blood samples during the period while eating meat harvested with lead bullets and when they are submitting blood samples while abstaining from lead-harvested meat so they will not be blinded. Animals will be shot in the thorax (Fig. 3) and the hunter will provide photographs of the shot location. Hunters should process the animal according to their standard practice, including making of minced meat.

Meals can be prepared at individual discretion; however, the meal must include at least 85 g of minced meat per test subject. This is equivalent to ¾ cup of minced meat and is considered a standard serving. Minced meat is used to reduce variability and allow easy determination of meal portion size. This value was used as the basis of the sample size calculation.

### 2.3 Outcome variables

The primary outcome variable for the study is blood lead levels and the secondary outcomes include: compliance with study protocol; measures of complete blood count; mass of lead projectile; and number of shots fired.

### 2.4 Inclusion and exclusion criteria

To be included in this study, participants must be over 18 years old and eat minced meat from the animals they harvest. Exclusion criteria are listed in Table 1.

**Table 1:** Exclusion criteria for the study include any activities that may increase the basal blood lead level.

Smoker
Any type of kidney dysfunction
Any mineralising tissue fracture
Pregnant women
Menopausal women
Individuals working in the following industries that may entail lead exposure:
a) lead–acid battery manufacture
b) lead smelting
c) non-ferrous smelting and casting (e.g. brass)
d) steel scrap smelting
e) scrap lead metal handling
f) cutting/welding steel scrap
g) machining or polishing lead-containing alloys
h) plastic production (where lead compounds are used as stabilisers)
i) demolition
j) lead soldering
k) plastic recycling
l) panel beating
m) paint removal
n) sandblasting
o) leadlight window manufacture
p) lead casting, e.g. fishing weights, toy soldiers
q) radiator repair,
r) car exhaust repair and engine reconditioning (for older makes and models of vehicles)
s) jewellery (silver) production
t) shooting range

### 2.5 Study procedures

Specifically, the trial will be executed in four steps:

1. Education and outreach

**Fig. 4:**
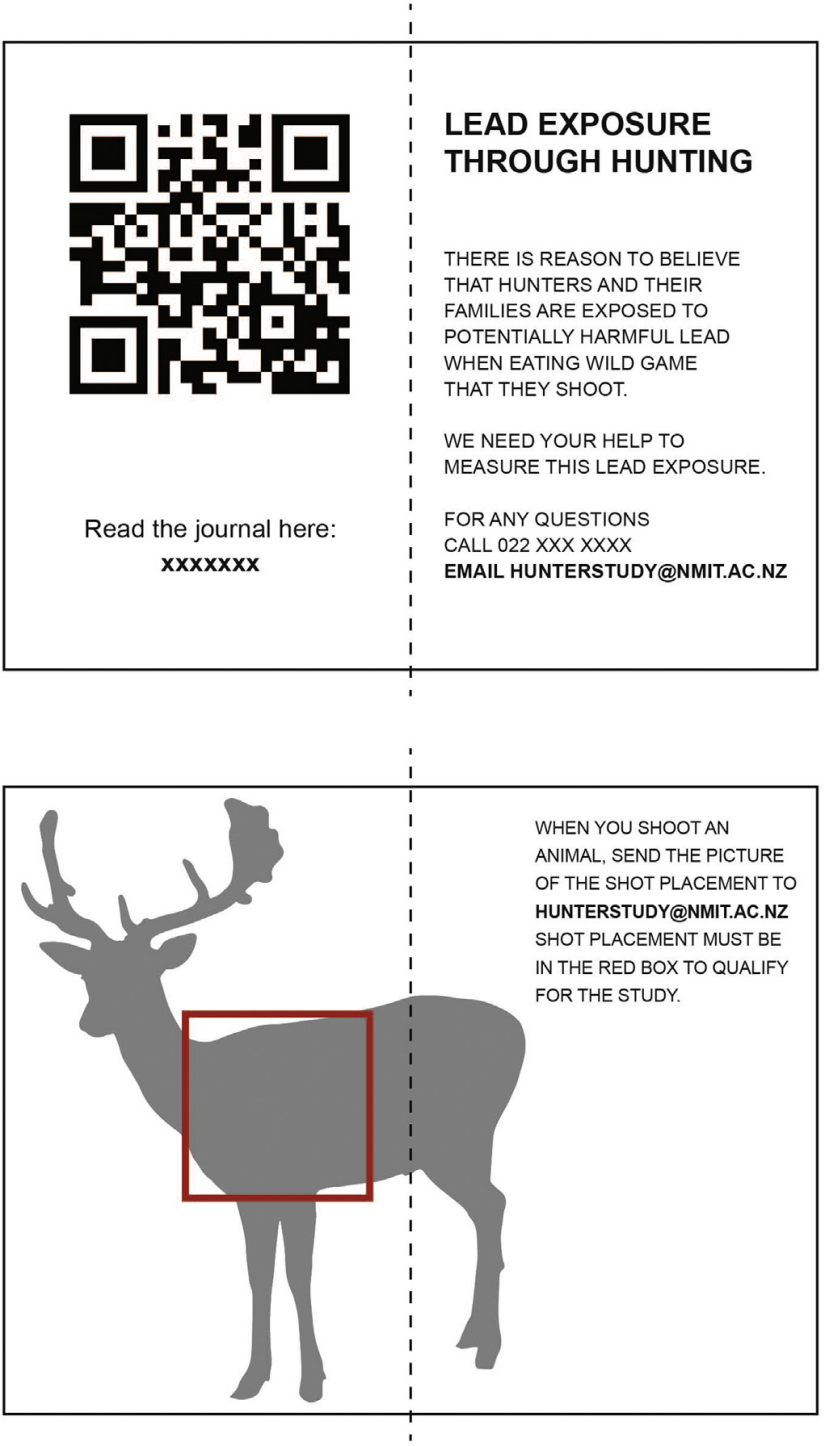
**Resource created for hunters interested in participating in the study.** For outreach and to facilitate participant recruitment, bi-fold business-card sized information sheets that can be kept with the potential participant's firearms license are being created. The front and the back of the resource is shown.

Hunters will be invited to participate in this study through clubs and professional organizations as well as through social media. There will be a documentary-style video trailer used as an outreach tool. An overview of the rationale for the trial as well as the study protocol will be shared at each of the club meetings. Business-card-size information resources will be handed out with contact and background information (Fig. 4). Additionally, the link to the online video describing the study will be emailed to hunters through the clubs.

It will be explained to hunters that after they shoot an animal that they must take a photograph of the animal to document shot placement. The photograph will be at least 72dpi resolution and clearly identify the location the animal was shot. The hunters must provide the weight of their bullet (brand if possible) and number of shots used to harvest the animal. This step is important to verify shot placement, and also to confirm the ability of the prospective participant to follow the directions for the study. The pictures indicating shot placement will be emailed to hunterstudy@nmit.ac.nz with the hunter contact information and a time available for a phone call. All hunters that send pictures will receive a phone call from the study coordinator.

2. Consent to enrol in study

Hunters that successfully email photographs of the harvested animals with the required information will be interviewed by the study coordinator for enrollment in the study. Enrollment in the study will occur in two steps. First, a telephone call will explain to the participant the consent form (see supplemental data) and answer questions the participant has regarding the study. The participant will be asked approximately how many times per week they eat lead-shot animals and if they are willing to abstain from eating lead-shot meat for 9, 18, or 27 days. Second, if the participant is still interested in participating, a consent form will be mailed to the participant and a time will be scheduled for a subsequent telephone call to allow formal consenting of the participant. Preliminary discussions with hunters have determined that a majority would be willing to abstain from eating wild-shot meat for one month for the study. However, if hunters decline to participate based on the length of abstaining from eating wild meat, they will be offered to still participate and abstain from eating lead-shot meat for 9 days (or the maximum they are willing to abstain). The consent form package will include a postage-paid envelope.

Formal consenting for the trial will occur via telephone and the study coordinator will work through the form with the participant. Once the pages have all been reviewed the participant will sign the consent form and return the form by mail.

3. Collection of study data

Hunters will be instructed to prepare their game in the usual manner. Typically, this involves mincing meat from the front legs of the animals and processing the rear quarters of the animal into larger cuts of meat. The participants will be instructed to text the study coordinator when processing occurs and the study coordinator will confirm receipt of the text message.

Prior to the first meat being eaten from the lead-shot animal, a baseline blood lead level will be measured. When a meal is eaten from the harvested game that involves ≥85 g (∼3/4 cup) of minced meat per person, the participants will be instructed to text the approximate number of servings eaten and notify the study coordinator of having eaten the meal. Between 2 and 4 days after eating the meal, the participant will report to the clinical laboratory in their local area for blood draw and complete blood count measurement. Each day that the participants are eligible for a blood draw they will receive texts from the study coordinator. After completing the blood draw, the participants will text the study coordinator.

Once the data from the clinical laboratory have been collected the participants will be contacted by the study coordinator. The participants will be instructed to report to the clinical laboratory at 9, 18 and 27 days during the abstention period for blood lead measures. Participants will receive a weekly text to remind them about the study. At the clinical laboratory, participants will provide a blood sample and the clinical laboratory will measure lead blood levels and complete blood count according to the standard clinical protocol. These values will be reported to the study coordinator. The study coordinator will maintain records of the results as described in Data Handling and Record Keeping.

During the study the participants will be requested to provide information on any activities that week that may have exposed them to lead such as reloading ammunition or any of the study exclusion criteria.

No biological samples will be retained.

4. Analyses and reporting of results

All data will be collected prospectively by the study coordinator and stored in a password-protected Excel spreadsheet for analysis. The data will be provided blinded to the Principal Investigator for analyses as described in the statistical methods section.

### 2.6 Primary outcome and sample size determination for the feasibility study

A two-tailed dependent *t*-test was utilized to perform a power analysis and determine sample size necessary for the study. A dependent *t*-test was used for the power analysis in order to ensure sample size would not be underestimated. To address correlation and the nature of longitudinal data, a conditional linear effects model will then be utilized to determine differences in the pattern of mean blood lead level over time. A sample size calculation was performed (SigmaPlot 12.5; Systat) and the following values were assigned based on previous literature and clinical logic: Expected Difference in Means: 0.074; Expected Standard Deviation: 0.19; Number of groups: 2; Desired Power: 0.8; Alpha: 0.05. The expected mean difference is derived from preclinical data [17] and 7 day washout. This calculation resulted in a sample size calculation of 54 data points in each abstention cohort.

We expect 25% attrition from the study and therefore we will be required to recruit a total of 68 participants.

### 2.7 Statistical methods and analysis of feasibility study

All data will be summarized using descriptive statistics (i.e. mean ± SEM). Data will be tested for normal distribution using a QQ plot and the Shapiro-Wilk test. Visualization and assessment of normality will be achieved through creation of histograms and boxplots. The primary dependent variable is mean blood lead level. In order to determine whether there is a difference in the pattern of mean blood lead level over time while accounting for lead exposure, mean blood lead level at baseline, 2–4 days, 9 days, 18 days and 27 days will be analysed using a conditional model that will account for the correlative nature of longitudinal data involving repeated measures taken from the same subject. This model is advantageous for a variety of reasons, including that this approach can handle unbalanced data (missing data at various time points), as well as continuous and discrete predictors. This model will also be able to model covariance structure which is important when handling correlated data. A random intercept will be utilized to account for natural variation in baseline blood lead measurements among hunters. Predictors will include the grouping variable of lead vs. lead-free projectile, time and the interaction of group and time. The interaction term will allow the investigators to identify whether blood lead level changes over time, thereby determining the most appropriate wash-out period. Other factors, such as any exposure to lead ammunition and isotope analysis may be included as factors in the analysis.

Categorical variables will be analyzed using chi-square tests. Variables including (agree to participate in study/decline to participate because of request to abstain/not because of request to abstain) will be used to determine the feasible length of abstaining from eating wild-shot meat. This value is important in determining the washout period for the interventional study. Adjustments using Fisher exact tests will be utilized to account for any cell counts that are 5 or less.

### 2.8 Statistical methods and analysis of interventional study

All data will be summarized using descriptive statistics (i.e. mean ± SEM). Data will be tested for normal distribution using a QQ plot and the Shapiro-Wilk test. Visualization and assessment of normality will be achieved through creation of histograms and boxplots. To determine differences in mean blood lead level over time in hunters who consume harvested meat with lead projectiles versus those with lead-free projectiles, a conditional linear effects model will also be utilized. This model will be similar to the model described in the feasibility analysis. A random intercept will be utilized to account for natural variation in baseline blood lead measurements among hunters. Predictors will include the grouping variable of lead vs. lead-free projectile, time and the interaction of group and time. Since this study is a cross-sectional study design, sequence of cohort (lead vs. lead-free) will also be adjusted for in the analysis. Other factors, such as any exposure to lead ammunition and isotope analysis may also be included as factors in the analysis. Two to three models will be assessed for fit, including a random intercept model, a linear random slope model and a quadratic random slope model if deemed appropriate. Model fit will be assessed using residual diagnostics and visuals such as scatter plots, interaction plots and time plots. Akaike's information criterion (AIC) will also be used to compare model fit. A variogram will be produced to aid in the appropriate identification of the variance-covariance structure and ensure that any unexplained autocorrelation is identified.

### 2.9 Missing data

To limit bias, this study will be guided and analyzed under the intention to treat principle, in that all participants will remain in the analysis regardless of missing data. The restricted maximum likelihood (REML) will facilitate the proper handling of unbalanced data or missing data, as well as mistimed measurements, so as to use the collected data in the most efficient way.

### 2.10 Ethics and dissemination

All data will be collected and managed by the clinical study coordinator. The data will be kept in a password protected database and a key to participant identifying information will be kept in a separate password protected file.

Blood draws will be collected by an independent contract clinical laboratory (MedLab). Blood samples will be analyzed for lead and complete blood count will be performed by the contract laboratory. Those blood data and time of the blood draw will be kept in the anonymized database. If requested by participants, their lead levels will be shared with them. In the event that the participant lead levels exceed 10 μg/dl (the standard level of concern in Australia and New Zealand) participants will be notified immediately with the recommendation to contact their general practitioner. These participants will not be withdrawn from the study.

Consent forms will be maintained in an alternate password-protected database with identifying information. Photographs of the animal harvested will be maintained in the anonymized database. All consented participants will be texted weekly and called monthly to determine if they have had a meal of harvested meat that week and if they have reported for the blood draw. Participant confidentiality will be maintained through a password protected database with a separate key to identify the anonymized data. Records of the study will be retained for 10 years and anonymized data will be deposited in a publicly-available repository.

## 3 Expected results

**Fig. 5:**
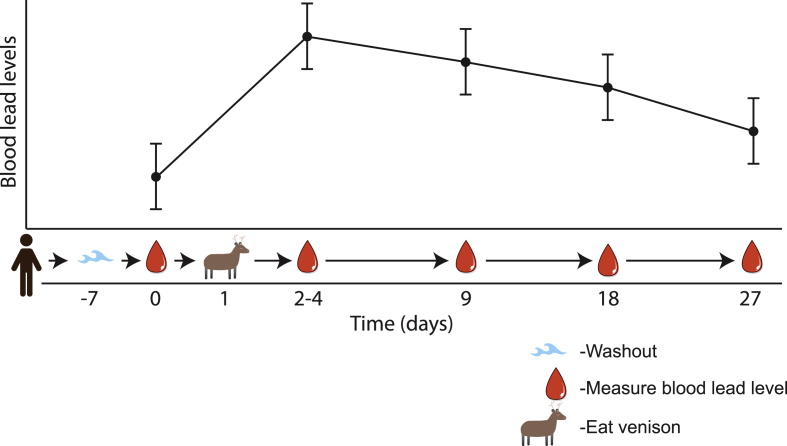
**Expected results from the blood lead level measures.** Following the washout period and baseline blood lead level measurement participants will eat their lead-shot deer. Two to four days after eating lead-shot deer be asked to abstain from eating lead-shot meat and blood lead levels will be assed at 9, 18 and 27 days. These blood lead levels will allow the sample size calculation, based on the three potential washout periods, for a subsequent interventional study to be calculated.

Fig. 5 illustrates expected results from this observational study. Specifically, post exposure to eating lead-harvested meat we will collect blood lead levels from individuals abstaining for 9, 18 and 27 days. Typically a study would require 5-half lives [21] for a washout period, however requesting hunters to abstain from eating lead-shot meat for 8180 days is not feasible. These data collected through this trial will allow us to calculate the necessary sample size and washout/run-in period for a subsequent cross over trial.

## 4 Discussion

Previous studies have established a correlation between blood lead levels and the use of lead ammunition to harvest wild game [22]. An interventional study is necessary to determine if this link between the use of lead ammunition and blood lead levels is causal. This feasibility study establishes the foundations required to conduct the necessary blinded, controlled study. The goal of this feasibility study is to determine the sample sizes necessary to determine a difference with the various washout periods.

It has been suggested that for this feasibility study including another group of participants using lead-free projectiles and randomizing these participants to the use of use lead-containing or lead-free projectiles would provide a more robust analysis. This type of interventional crossover study would conclusively determine causality and is exactly the interventional study that we propose to conduct after this current feasibility study. The feasibility study is critical to determine if this larger, more expensive, study is possible and to perform the appropriate sample size calculation.

For the subsequent crossover study (Fig. 2), a simple two period two treatment cross-over trial would be utilized. . This would allow comparison between mean blood lead levels in hunters randomized to two groups: those who eat wild game harvested with lead-projectiles versus those who do not utilize lead-projectiles. All hunters would undergo an initial wash-out period and then have baseline blood lead level recorded. Hunters would then eat the harvested meat and blood lead levels would be recorded 2–4 days after eating the self-harvested meat, at least 3 times during the year. After one year, the hunter would cross over to the other type of projectile. This case crossover approach would allow a factorial analysis of variance (ANOVA) to be conducted to determine differences in blood lead levels, while accounting for factors such as period and group (lead projectile vs. lead-free projectile).

As there is no safe level of lead exposure, determining if individuals eating wild game are exposed to lead through the self-harvested meat is an important global public health concern.

## Declarations

### Ethics approval

This study has been approved by the New Zealand Health and Disabilities Ethics Committees (ref 16/CEN/49).

### Availability of data and materials

Data supporting this study is included as supplemental material.

### Competing interests

The authors declare that they have no competing interests.

### Funding statement

This research received no specific grant from any funding agency in the public, commercial or not-for-profit sectors.

### Author contributions

EJB and GJP conceived and design the study. BAB provided critical revisions to the study design. EJB drafted the manuscript. LMM provided statistical design for the study. LMM, GJP and BAB provided critical revision of the manuscript. KB provided the graphic designs and figures. All authors approve the final version of the manuscript.
