# Supplementary material for: A prospective observational study assessing the feasibility of measuring blood lead levels in New Zealand hunters eating meat harvested with lead projectiles
Source: Contemp Clin Trials Commun. 2017 Feb 8;5:137–43. doi: 10.1016/j.conctc.2017.02.002 (PMC5936706; doi:10.1016/j.conctc.2017.02.002)
Supplement: Supplementary file 1 [file mmc1.docx]

**Participant Information Sheet**

***A prospective observational case-crossover study assessing the feasibility of measuring blood lead levels in New Zealand hunters using lead projectiles***

**Study Contact:** Dr. Eric Buenz

[Eric.Buenz@nmit.ac.nz](mailto:Eric.Buenz@nmit.ac.nz)

Dr. Gareth Parry

[Gareth.Parry@nmit.ac.nz](mailto:Gareth.Parry@nmit.ac.nz)

You are being asked to take part in a study evaluating lead exposure in hunters through eating wild game. Please take the time to read this information sheet carefully. You may wish to discuss this information with family, whānau or healthcare providers. Feel free to do this. We are happy to answer any questions about the study.

This Participant Information Sheet will help you decide if you’d like to take part. It sets out why we are doing the study, what your participation would involve, what the benefits and risks to you might be, and what would happen after the study ends. We will go through this information with you and answer any questions you may have. We expect this will take about 5 minutes.

Whether or not you take part is your choice. If you don’t want to take part, you don’t have to give a reason, and it won’t affect the care you receive. If you do want to take part now, but change your mind later, you can pull out of the study at any time.

If you agree to take part in this study, you will be asked to sign the Consent Form on the last page of this document. You will be given a copy of both the Participant Information Sheet and the Consent Form to keep.

This document is 4 pages long, including the Consent Form. Please make sure you have all the pages.

**Why are we doing the study?**

There is no safe level of lead exposure and most bullets are made of lead. When wild game is shot with lead bullets, hundreds of pieces of shrapnel are scattered throughout the animal. It isn’t known if eating these pieces of shrapnel causes people to have raised lead levels in their body. This study will measure if hunters that have recently eaten animals shot with lead projectiles have higher lead levels in their body compared to when they have not recently eaten animals shot with lead bullets.

There is no funding available to assist participants to take part, so everyone will need to be able to attend tests and appointments required for the study There will be no cost to participants for the blood testing or participating in the study.

**Who is eligible for the study?**

Hunters over 18 years old that hunt with lead bullets and eat minced meat from the animals that they harvest. People who work in industries with occupational lead exposure cannot participate in this study.


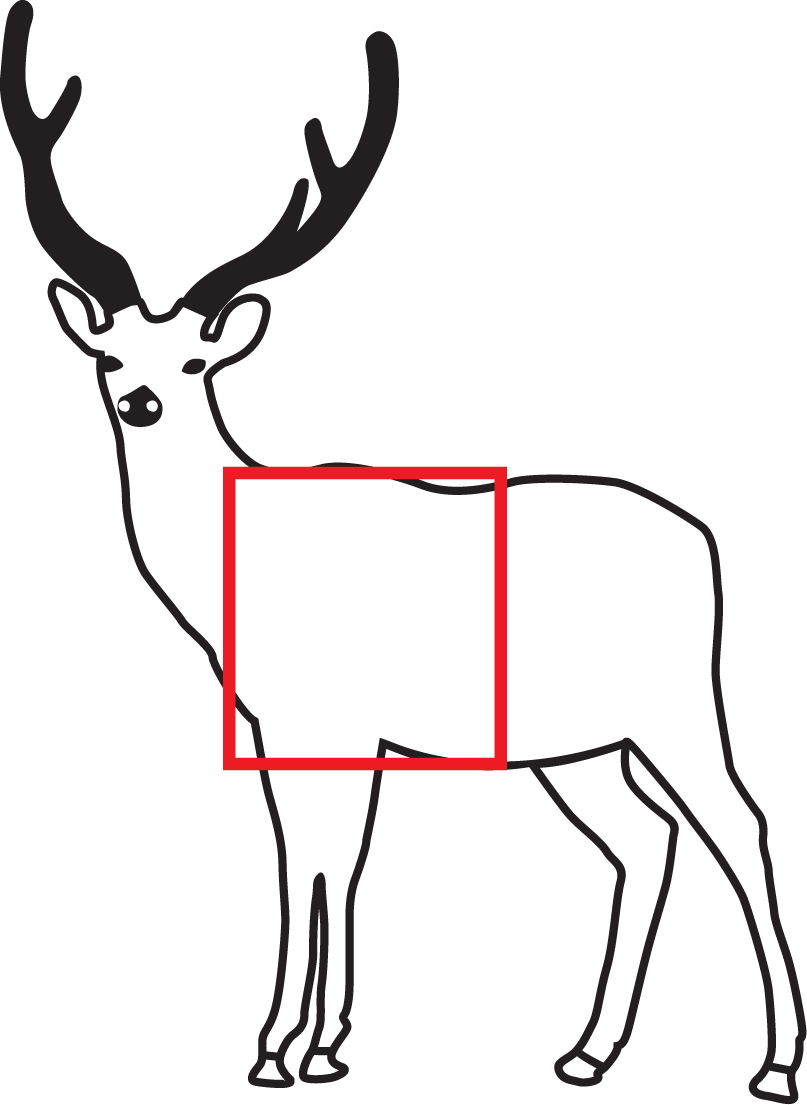


Figure 1. Animals harvested for this study will be shot in the area indicated by a red box.

**What would your participation involve?**

The study will last for up to one year and you will give two blood samples at MedLab. One sample will be taken 2-4 days after you eat minced meat harvested with lead bullets. A different sample of blood will be taken when you haven’t eaten wild game harvested with lead bullets for at least one week. You are welcome to have a family or whānau member or friend with you if you wish.

You will have to shoot a deer in the area shown and email a picture of your shot placement to the study coordinator.

**What are the possible benefits and risks to you of participating?**

The benefit of this study is that you are helping answer an important medical question. Also, after the study is completed we will tell you if your lead level was higher when you ate meat harvested with lead bullets. The risk of the study is associated with the blood draw. The risks involved with drawing blood from a vein include momentary discomfort at the site of the blood draw, possible bruising, redness, and swelling around the site, bleeding at the site, feeling of lightheadedness when the blood is drawn, and rarely, an infection at the site of the blood draw. If your lead levels are high you will be referred to your doctor.

**What would happen if you were injured in the study?**

If you were injured in this study, which is unlikely, you may be eligible for compensation from ACC just as you would be if you were injured in an accident at work or at home.

**What are my rights?**

Participation in the study is your choice. You are free to decline to participate, or withdraw from the study at any time. This will in no way affect your usual care you receive.

You have the right to access any information or results collected as part of the study. All information collected about you will be linked to a trial number and not linked to your clinical file. When the results are published there will be no information that could allow you to be recognized. Only the primary investigator will be able to link you to your trial number.

**What will happen after the study ends, or if I pull out?**

Study data will be stored securely for 10 years. This will not be linked to your name, but a study number. It will then be destroyed by confidential document destruction.

The results will be analysed and a research report written. This will be published in a medical journal and presented at a conference. You will receive a summary of the results by post.

**Where can you go for more information about the study, or to raise concerns or complaints?**

If you have any questions about the study at any stage, you can contact:

*Dr. Eric Buenz, Principal Investigator*

[Eric.Buenz@nmit.ac.nz](mailto:Eric.Buenz@nmit.ac.nz)

*022 513 5791*

*Dr. Gareth Parry, Lead Clinician*

*Gareth.Parry@nmit.ac.nz*

*021 456 738*

If you want to talk to someone who isn’t involved with the study, you can contact an independent health and disability advocate on:

Phone: 0800 555 050
Fax: 0800 2 SUPPORT (0800 2787 7678)
Email: [advocacy@hdc.org.nz](mailto:advocacy@hdc.org.nz)

If you would like to talk to Whanau Care Services for support, you can contact them through:

Phone: 03) 546 1415

**If you need an INTERPRETER please tell us**

| **Consent Form** |  |
| --- | --- |

**Declaration by participant:**

I have read, or have had read to me in my first language, and I understand the Participant Information Sheet. I have had the opportunity to ask questions and I am satisfied with the answers I have received.

I freely agree to participate in this study.

I have been given a copy of the Participant Information Sheet and Consent Form to keep.

| Participant’s name: | |
| --- | --- |
| Signature: | Date: |

**Declaration by member of research team:**

I have given a verbal explanation of the research project to the participant, and have answered the participant’s questions about it.

I believe that the participant understands the study and has given informed consent to participate.

| Researcher’s name: | |
| --- | --- |
| Signature: | Date: |
